# Clinical and Epidemiological Implications of 24‐Hour Ambulatory Blood Pressure Monitoring for the Diagnosis of Hypertension in Kenyan Adults: A Population‐Based Study

**DOI:** 10.1161/JAHA.116.004797

**Published:** 2016-12-15

**Authors:** Anthony O. Etyang, Ben Warne, Sailoki Kapesa, Kenneth Munge, Evasius Bauni, J. Kennedy Cruickshank, Liam Smeeth, J. Anthony G. Scott

**Affiliations:** ^1^ KEMRI‐Wellcome Trust Research ProgrammeKilifiKenya; ^2^ London School of Hygiene and Tropical MedicineLondonUnited Kingdom; ^3^ King's CollegeLondonUnited Kingdom

**Keywords:** ambulatory blood pressure monitoring, diagnostic accuracy, hypertension, masked hypertension, sub‐Saharan Africa, white coat hypertension, High Blood Pressure, Hypertension, Diagnostic Testing

## Abstract

**Background:**

The clinical and epidemiological implications of using ambulatory blood pressure monitoring (ABPM) for the diagnosis of hypertension have not been studied at a population level in sub‐Saharan Africa. We examined the impact of ABPM use among Kenyan adults.

**Methods and Results:**

We performed a nested case–control study of diagnostic accuracy. We selected an age‐stratified random sample of 1248 adults from the list of residents of the Kilifi Health and Demographic Surveillance System in Kenya. All participants underwent a screening blood pressure (BP) measurement. All those with screening BP ≥140/90 mm Hg and a random subset of those with screening BP <140/90 mm Hg were invited to undergo ABPM. Based on the 2 tests, participants were categorized as sustained hypertensive, masked hypertensive, “white coat” hypertensive, or normotensive. Analyses were weighted by the probability of undergoing ABPM. Screening BP ≥140/90 mm Hg was present in 359 of 986 participants, translating to a crude population prevalence of 23.1% (95% CI 16.5–31.5%). Age standardized prevalence of screening BP ≥140/90 mm Hg was 26.5% (95% CI 19.3–35.6%). On ABPM, 186 of 415 participants were confirmed to be hypertensive, with crude prevalence of 15.6% (95% CI 9.4–23.1%) and age‐standardized prevalence of 17.1% (95% CI 11.0–24.4%). Age‐standardized prevalence of masked and white coat hypertension were 7.6% (95% CI 2.8–13.7%) and 3.8% (95% CI 1.7–6.1%), respectively. The sensitivity and specificity of screening BP measurements were 80% (95% CI 73–86%) and 84% (95% CI 79–88%), respectively. BP indices and validity measures showed strong age‐related trends.

**Conclusions:**

Screening BP measurement significantly overestimated hypertension prevalence while failing to identify ≈50% of true hypertension diagnosed by ABPM. Our findings suggest significant clinical and epidemiological benefits of ABPM use for diagnosing hypertension in Kenyan adults.

## Introduction

Across sub‐Saharan Africa (sSA), numerous studies have been conducted to estimate the prevalence of hypertension in various settings.^1^, ^2^ These data are necessary for planning public health activities, especially given the projected increase in the burden of noncommunicable disease in the region.^3^ Nevertheless, a substantial amount of communicable disease remains, increasing the need for accurate data to guide resource allocation among competing health priorities.^4^

The measurement of blood pressure (BP) is subject to multiple sources of variation^5^ that will have an impact on both the epidemiology and the clinical management of hypertension. Measurement error arising from technical problems has been minimized by using validated criteria for BP monitors.^6^ It is possible to minimize observer error in reading BP values by using automated devices and following a defined measurement procedure. Despite such improvements, these protocols provide only a momentary assessment of BP, which can be influenced by a variety of environmental and psychological factors.^7^ Ambulatory BP monitoring (ABPM) reduces this limitation and is increasingly used for both clinical and epidemiological purposes.^7^, ^8^ In high‐income settings, ABPM is a significantly better predictor of future cardiovascular risk than office or home measurement.^7^ It is also the only way to detect abnormal nocturnal dipping patterns that are an independent risk factor for future cardiovascular events^9^ and that appear to be more common in populations of African descent.^10^, ^11^ In addition, ABPM enables the measurement of the ambulatory arterial stiffness index, an independent predictor of cardiovascular outcomes, especially stroke.^12^

The use of ABPM facilitates better prescription patterns among established hypertensive patients and helps identify patients with masked hypertension and those with abnormal dipping patterns who would otherwise be missed when using casual (standard) BP measurement methods. The increased diagnostic performance of ABPM makes it cost effective in both primary and specialist care in developed‐world settings.^13^, ^14^ The high initial cost of ABPM devices has precluded their widespread use in low‐income settings in sSA, and there is little information to assess the potential clinical and public health benefits of ABPM in such settings.

We conducted Shinikizo la Damu (ShinDa), a population‐based study, in rural coastal Kenya to determine 24‐hour BP profiles in adults and to compare these with parameters derived from screening BP measurements.

## Methods

This study was conducted from April 2013 to May 2014 in the Kilifi Health and Demographic Surveillance System (KHDSS) located along the coast of Kenya. The 900 km^2^ covered by the KHDSS and a population of 300 000 people makes it one of the largest demographic surveillance areas in Africa both in terms of area and population.^15^ Within the KHDSS, the total fertility rate (4.73), the crude birth rate (34.7 per 1000 annually), the population growth rate (2.79% per year), and the proportion of the population aged <15 years (49%) are similar to rates across Kenya. However, the mortality ratio for children aged <5 years (41 per 1000 births) and HIV prevalence (4.9% among antenatal clinic attendees) are lower than the national averages (74 per 1000 births and 8.0%, respectively).^16^ We have previously documented a double burden of infectious and noncommunicable diseases in the area, similar to other parts of the developing world.^17^

We selected an age‐stratified random sample of 1248 adults from the list of KHDSS residents. The sample size was designed to generate a population‐level estimate of the true prevalence of hypertension with confidence intervals of approximately ±5%. We previously documented a significant distance‐related bias in presentation to the hospital in the study area.^17^ To minimize this bias, study procedures were carried out at the participants’ homesteads. Trained staff visited all participants who were selected to participate in the study at their homes. A total of 3 attempts were made at finding a selected participant before concluding that the participant could not be found. No replacement was done for participants who could not be found. Women who reported that they were pregnant were excluded from the study.

We used a nested case–control diagnostic accuracy study design in which all participants with elevated screening BP and a random subset of those with normal screening BP were invited to undergo ABPM.^18^ We weighted analyses to account for differential probability of undergoing ABPM based on the result of screening BP measurement.^18^ We aimed to have 24‐hour ABPM performed on equal numbers of participants with and without elevated screening measurements (≥140/90 mm Hg), assuming that the prevalence of elevated screening BP would be ≈30%.

All participants were first asked whether they had a previous diagnosis of hypertension and whether they were on antihypertensive medication. We then took a screening BP measurement using a validated Omron M10‐IT BP monitor. An appropriately sized cuff was placed on the nondominant arm after the participant was seated for at least 5 minutes. Three BP measurements were taken over a 5‐minute period, and the mean of the last 2 measurements was recorded as the screening BP value. All participants whose screening BP was ≥140 and/or 90 mm Hg were invited to undergo 24‐hour ABPM. A random sample of 292 (≈30%) of patients with screening BP <140/90 mm Hg were invited for ABPM, and analyses were weighted (see the “Statistical Methods” section) to account for this. We used validated Omron M24/7 ambulatory BP monitors for the 24‐hour ABPM measurements.^19^ The monitors were programmed to take BP measurements every 20 to 30 minutes from 6 am to 10 pm and every 40 minutes from 10 pm to 6 am. ABPM was performed within 1 week of the screening BP measurements in all cases. The same field staff conducted the screening and assessed ABPM measurements and was not blinded to any of the results.

A subset of 200 participants was selected at random from the original sample of 1248 and asked to participate in additional investigations. We first measured their weight and height using a validated Seca 874 flat scale and a portable stadiometer (Seca 213), respectively. We then requested participants to provide a spot urine sample and a 24‐hour urine sample for determination of sodium, potassium, albumin, and creatinine levels.

### Statistical Methods

ABPM data were excluded from the analyses if they failed the following criteria, as specified by the European Society of Hypertension: minimum 20 daytime and minimum 7 nighttime readings, for which *day* was defined as 9 am to 9 pm and *night* was defined as 1 am to 6 am.^7^ The same time periods were used to determine average daytime and nighttime BPs and to evaluate dipping status. Time weighting was applied in calculating average BP values for all time periods.^20^

We defined *screen‐positive* participants as those whose last 2 screening BP measurements had a mean ≥140/90 mm Hg, and *confirmed hypertensive* participants were defined as those who met any of the following 3 criteria: 24‐hour BP average ≥130/80 mm Hg, isolated daytime hypertension (daytime BP average ≥135/85 mm Hg and nighttime BP average <120/70 mm Hg), and isolated nocturnal hypertension (nighttime BP average ≥120/70 mm Hg and daytime BP average <135/85 mm Hg).^7^

Validity measures were computed using data from participants who were not taking antihypertensive medications. We categorized these participants using the combination of screening BP measurements and ABPM into 4 groups: sustained hypertensive (screen‐positive and confirmed hypertensive on ABPM), “white coat” hypertensive (screen‐positive but not confirmed hypertensive on ABPM), masked hypertensive (screen‐negative but confirmed hypertensive on ABPM), or normotensive (screen‐negative and not confirmed hypertensive on ABPM).^21^ The 4 categories were used to compute age‐stratified measures of validity of the screening BP measurements with ABPM as the reference standard.^22^, ^23^ All summary measures were weighted according to the probability of investigation with ABPM in the design.^24^ Confidence intervals for these measures were obtained using the recommended bootstrap procedure with 1000 replications.^24^

Among participants who reported that they were taking antihypertensive medications, those who met the criteria for white coat hypertension were labeled as having *pseudoresistant* hypertension,^7^ whereas those who met the criteria for masked hypertension were labeled as *masked uncontrolled* hypertensive.^7^

Dipping status was defined using ABPM data only, using previously described methods.^25^

We computed 2 additional indices using the ABPM data—24‐hour pulse pressure (the mean of the differences between systolic and diastolic BP values) and the ambulatory arterial stiffness index—using previously published methods.^26^

We calculated the local prevalence of each index by weighting the age‐specific proportions by the age structure of the Kilifi population. Age standardization for all summary population parameters was performed using weights derived from the World Health Organization standard population.^27^ Summary statistics included means, medians, proportions, and rates, as appropriate.

All analyses were conducted using Stata version 12 software (StataCorp).

The Kenya Medical Research Institute's ethics review committee approved the study, and all participants provided written informed consent.

## Results

**Figure 1:**
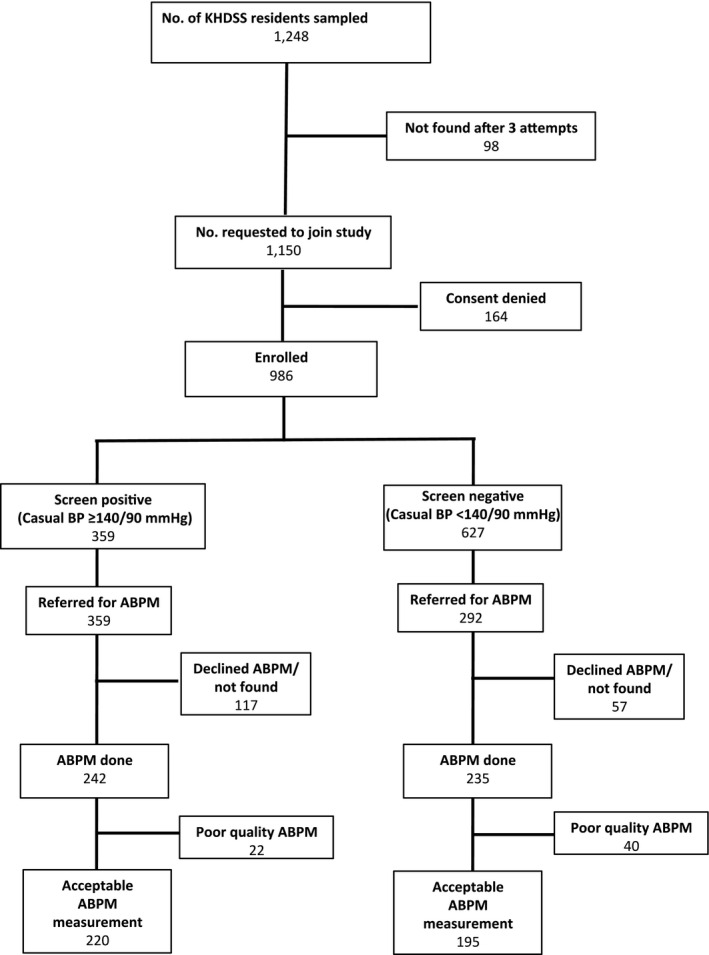
Study recruitment profile. ABPM indicates ambulatory blood pressure monitoring; BP, blood pressure; KHDSS, Kilifi Health and Demographic Surveillance System.

Of the 1248 patients selected to participate in the study, 1150 (92%) were found at home and invited to participate in the study. Of these, 986 (86%) gave consent and underwent screening BP measurement (Figure 1). Of these, 359 (36%) participants were screen‐positive for hypertension (screening BP ≥140/90 mm Hg). All 359 screen‐positive and 292 screen‐negative participants were invited to undergo ABPM. Of all those invited to undergo ABPM, 477 (73%) actually underwent it. Of the 200 participants selected to have urine electrolyte and anthropometric measurements, 164 (82%) did so.

Table 1 compares the characteristics of all 986 participants who had screening BP measurements, the subset of 651 selected to undergo ABPM, and the 477 who actually underwent ABPM. The group that had ABPM performed had higher screening systolic BP (+5 mm Hg, 95% CI 4.2–5.9) and were older (+3.9 years, 95% CI 3.4–4.3) compared with the entire group of participants selected for the study. There were no significant differences between those selected to undergo ABPM (n=651) and those who actually underwent the procedure (n=477).

**Table 1:** Characteristics of Study Participants

	All (N=986)	Selected to Undergo ABPM (n=651)	ABPM Performed (n=477)
Women, n (%)	588 (60)	400 (61)	302 (63)
Age in years, mean (SD)	50 (19)	53 (18)	54 (18)^a^
Screening BP in mm Hg, mean (SD)			
Systolic	134 (26)	142 (28)	139 (28)^a^
Diastolic	81 (13)	84 (15)	82 (15)
BMI, kg/m^2^ (median, IQR)^b^	21 (19–24)	21 (19–24)	21 (19–23)
Na excretion, mmol/24 h (median, IQR)^b^	63 (47–92)	62 (47–90)	61 (47–80)
K excretion, mmol/24 h (median, IQR)^b^	25 (18–38)	25 (19–38)	25 (19–38)
Spot urine albumin:creatinine ratio, mg/mg (median, IQR)^b^	0.02 (0.01–0.04)	0.02 (0.01–0.04)	0.02 (0.01–0.04)
On medication for hypertension, n (%)	22 (2)	16 (2)	7 (1)

ABPM indicates ambulatory blood pressure monitoring; BMI, body mass index; BP, blood pressure; IQR, interquartile range.

a *P*<0.001 comparison between all screened participants (N=986) and those who underwent ABPM (n=477).

b BMI, Na, K, urine albumin:creatinine ratio based on 164 participants.

Of the 477 participants who underwent ABPM, 415 (87%) had acceptable readings; unacceptable readings (<20 daytime and/or <7 nighttime) were significantly more common among screen‐negative than screen‐positive participants (17% versus 11%, *P*=0.036). Data from participants with unacceptable ABPM readings were dropped from further analyses.

Six of the 415 participants with acceptable ABPM recordings were on antihypertensive medication.

### Relationship Between Screening BP Measurements and ABPM‐Derived Measures

**Figure 2:**
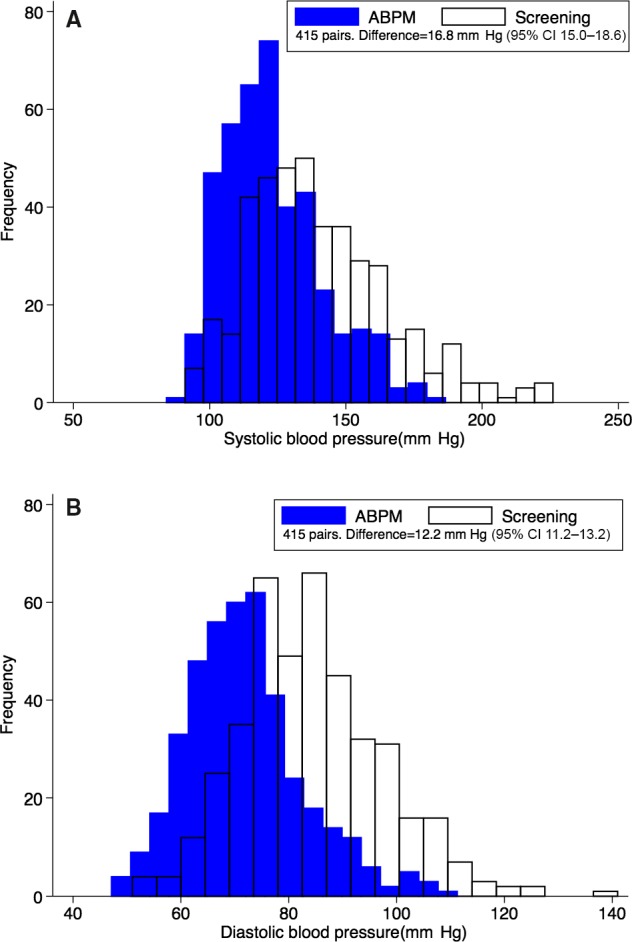
Comparison of screening and ABPM blood pressure distributions. A, Systolic. B, Diastolic. ABPM indicates ambulatory blood pressure monitoring.

Among the 415 participants who had both screening and 24‐hour ABPM measurements, mean screening systolic and diastolic BPs were 140 mm Hg (95% CI 138–143) and 84 mm Hg (95% CI 83–85), respectively. Corresponding mean 24‐hour ABPM systolic and diastolic BPs were 123 mm Hg (95% CI 121–125) and 72 mm Hg (95% CI 71–73). The average difference between mean systolic screening and 24‐hour ABPM values was 16.8 mm Hg (95% CI 15–18.6). Mean screening diastolic BPs were 12.2 mm Hg (95% CI 11.2–13.2) higher than corresponding 24‐hour ABPM values (Figure 2).

Age‐standardized mean screening systolic and diastolic BPs for the population in Kilifi were 128 mm Hg (95% CI 102–162) and 79 mm Hg (95% CI 62–101), respectively. The 24‐hour mean systolic and diastolic BPs for the population were 117 mm Hg (95% CI 114–120) and 70 mm Hg (95% CI 68–72), respectively.

ABPM‐derived mean BPs were lower than those obtained using screening measurement methods for all age groups (Table 2). The relationship between 24‐hour ABPM‐derived parameters (except for ambulatory arterial stiffness index and diastolic BPs) and age approximated a *J* shape. Mean ambulatory arterial stiffness index and pulse pressure increased linearly with age (0.03 [95% CI 0.02–0.05] and 3.3 [95% CI 1.5–5], respectively) per 10‐year increase in age. The difference between ABPM‐derived and screening BP values increased with age.

**Table 2:** Mean BP, Pulse Pressure and AASI, by Age, Derived From Screening BP and 24 Hour ABPM

Age Group, y	Mean Screening BP		24‐Hour ABPM Measures							
			Daytime Mean		Nighttime Mean		24‐Hour Mean		Mean Stiffness Indices	
	Systolic	Diastolic	Systolic	Diastolic	Systolic	Diastolic	Systolic	Diastolic	Pulse Pressure	AASI
18–29	120 (102–144)	76 (60–92)	118 (115–122)	71 (69–73)	107 (103–112)	60 (58–63)	115 (111–118)	68 (66–70)	47 (45–49)	0.31 (0.27–0.36)
30–39	120 (96–151)	77 (61–101)	112 (110–115)	71 (69–73)	101 (98–105)	60 (58–63)	109 (106–112)	68 (66–70)	41 (40–43)	0.38 (0.34–0.43)
40–49	128 (100–164)	81 (64–104)	119 (116–122)	74 (72–76)	106 (104–109)	63 (61–65)	115 (112–118)	70 (68–72)	45 (43–46)	0.41 (0.37–0.45)
50–59	135 (100–176)	83 (61–106)	125 (122–128)	76 (74–79)	114 (111–117)	66 (64–68)	122 (119–125)	73 (71–75)	49 (47–50)	0.41 (0.38–0.45)
60–69	147 (109–196)	85 (66–112)	131 (128–134)	77 (75–79)	122 (119–125)	68 (66–71)	127 (125–131)	74 (72–76)	53 (51–56)	0.49 (0.46–0.52)
70–79	153 (116–196)	84 (63–107)	132 (128–135)	72 (70–74)	129 (125–133)	66 (64–68)	131 (127–134)	70 (68–72)	60 (58–63)	0.53 (0.48–0.57)
≥80	156 (113–217)	86 (56–110)	137 (130–143)	75 (70–80)	129 (123–135)	68 (64–71)	134 (127–140)	72 (68–77)	62 (57–66)	0.52 (0.47–0.56)

Units for all measurements except AASI are in mm Hg. AASI is an index with no units. Statistics are mean and 95% CI. AASI indicates ambulatory arterial stiffness index; ABPM, ambulatory blood pressure monitoring; BP, blood pressure.

### Prevalence of Hypertension Using Casual BP Measurement and ABPM

All reported KHDSS population parameters are age standardized unless otherwise specified. The crude prevalence of hypertension in the KHDSS using screening measurements only was 23.1% (95% CI 16.5–31.5%). The crude prevalence of hypertension in the KHDSS using ABPM was 15.6% (95% CI 9.4–23.1%). Using only screening BP values, the reported age‐standardized prevalence of hypertension would have been 26.5% (95% CI 19.3–35.6%). The age‐standardized prevalence of true hypertension (as determined by ABPM) in the KHDSS was 17.1% (95% CI 11.0–24.4) (Table 2). There was a marked increase in prevalence of screen‐positive participants and true hypertension with increasing age (Table 3).

**Table 3:** Age‐Specific Prevalence of Hypertension Using Screening Measurement Method and ABPM

Age, y	Screen‐Positive	HTN^a^	Masked HTN^b^	White Coat HTN^c^	Nondipping Status^d^
18–29	11.0 (6.5–17.3)	10.9 (4.3–19.5)	10.9 (4.3–19.5)	2.9 (1.5–4.4)	6.5 (0.0–15.2)
30–39	13.1 (8.1–20.1)	12.5 (5.7–21.2)	11.5 (4.9–19.7)	1.0 (0.2–2.0)	9.8 (3.3–18.0)
40–49	26.8 (19.3–36.2)	11.0 (6.5–16.1)	4.5 (1.1–9.0)	3.7 (1.6–6.2)	5.3 (2.0–9.5)
50–59	43.8 (34.4–55.0)	20.2 (15.6–25.5)	2.8 (0.7–5.6)	7.0 (3.8–10.3)	6.8 (3.6–10.7)
60–69	55.4 (44.7–67.8)	33.4 (27.7–39.2)	3.1 (1.0–5.7)	3.9 (1.3–7.1)	12.1 (7.7–16.5)
70–79	65.0 (51.4–81.1)	47.1 (39.7–52.6)	2.7 (0.5–4.8)	10.0 (5.0–16.0)	24.2 (17.4–31.0)
≥80	68.8 (47.3–96.6)	46.9 (35.2–58.3)	3.9 (0.0–7.8)	8.6 (0.0–17.2)	12.2 (4.0–22.4)
All^e^	26.5 (19.3–35.6)	17.1 (11.0–24.4)	7.6 (2.8–13.7)	3.8 (1.7–6.1)	8.5 (3.1–15.3)

ABPM indicates ambulatory blood pressure monitoring; BP, blood pressure; HTN, hypertension.

a Hypertension defined according to European Society of Hypertension 2013 guidelines^7^: 24‐hour BP >130/80 mm Hg or daytime BP >135/85 mm Hg or nocturnal BP >120/70 mm Hg. Nighttime was defined as 1 am to 6 am. Daytime was defined as 9 am to 9 pm.^7^

b Masked HTN: casual BP <140/90 mm Hg but meets criteria for HTN on ABPM.^7^ Participants with masked HTN were included in the group with true HTN.

c White coat HTN: Casual BP >140/90 mm Hg but does not meet criteria for HTN on ABPM.^7^

d Nondipping status: ratio of average nighttime BP to average daytime BP ≥1.0.

e Summary prevalences are age standardized to the World Health Organization population.

**Figure 3:**
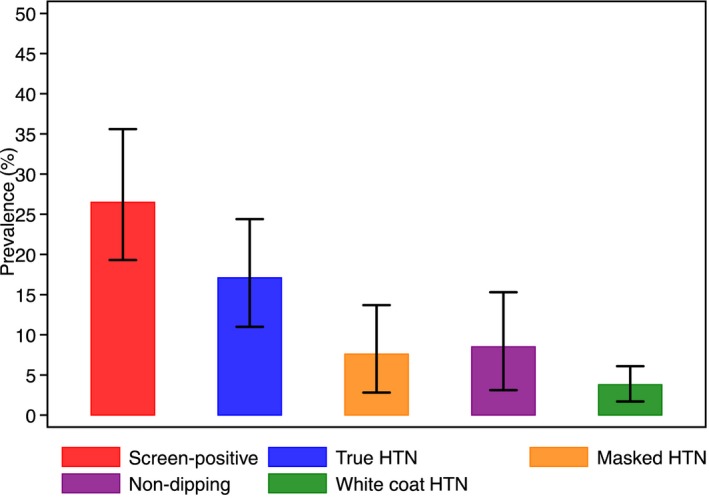
Population prevalence of different blood pressure indices. Data are derived from Table 3. HTN indicates hypertension.

The prevalence of masked hypertension overall was 7.6% (95% CI 2.8–13.7%), and this was inversely associated with increasing age. White coat hypertension was present in 3.8% (95% CI 1.7–6.1%) of the population, and its prevalence increased with age. The nondipping BP pattern was present in 8.5% (95% CI 3.1–15.3%) of the population, its prevalence being highest in the 70‐ to 79‐year age band. Figure 3 displays the standardized population prevalence of screen‐positive, true hypertensive, white coat hypertensive, masked hypertensive, and nondipping participants.

Two of the 6 participants on antihypertensive medication who underwent ABPM had pseudoresistant hypertension. No cases of masked uncontrolled hypertension were detected.

### Validity of Screening BP Measurements

The overall sensitivity and specificity of screening BP measurements for diagnosing hypertension in the population were 80% (95% CI 73–86%) and 84% (95% CI 79–88%), respectively. Sensitivity improved with increasing age, whereas specificity decreased (Table 4). Overall positive and negative predictive values were 80% (95% CI 74–85%) and 84% (95% CI 79–89%), respectively. Sensitivity and positive predictive value increased with age; specificity and negative predictive value decreased with age. Likelihood ratio positive was 4.9 (95% CI 3.7–6.8) and was highest in the 30‐ to 49‐year age group, although the confidence intervals were wide. Likelihood ratio negative overall was 0.2 (95% CI 0.2–0.3). No significant age‐related trend was observed in the likelihood ratio–negative values. Interval likelihood ratios based on quintiles of the screening systolic and diastolic BPs are displayed in Table 5. Screening BP measurements performed best in predicting true diagnostic category for participants with diastolic BPs of <80 mm Hg and systolic BP of 118 to 129 mm Hg.

**Table 4:** Validity Measures of Casual BP Method Compared to ABPM

Age Category, y	Sensitivity	Specificity	PPV	NPV	LR Positive	LR Negative
18–29	0.0 (0.0–0.0)	95.0 (90.5–98.0)	0.0 (0.0–0.0)	83.3 (69.7–96.1)	0.0 (0.0–0.0)	1.1 (1.0–1.1)
30–39	9.7 (2.5–24.9)	98.4 (96.5–99.6)	55.6 (23.6–88.9)	84.1 (73.2–93.3)	6.1 (1.3–31.5)	0.9 (0.8–1.0)
40–49	60.8 (35.8–88.7)	91.8 (85.4–96.6)	63.6 (45.1–83.3)	90.9 (80.5–97.7)	7.5 (3.4–20.8)	0.4 (0.1–0.7)
50–59	87.5 (74.8–96.9)	74.8 (62.0–86.0)	72.3 (59.5–84.5)	88.6 (76.1–97.1)	3.4 (2.2–6.3)	0.2 (0.0–0.3)
60–69	91.2 (83.4–97.0)	71.6 (53.3–87.4)	86.0 (75.9–94.3)	80.6 (64.5–92.9)	3.2 (1.9–7.0)	0.1 (0.0–0.3)
70–79	94.2 (88.1–98.9)	24.4 (8.5–49.2)	81.7 (70.4–90.9)	54.5 (25.0–85.7)	1.2 (1.0–1.8)	0.2 (0.0–0.8)
≥80	92.1 (81.5–100.0)	31.2 (0.0–100.0)	84.2 (64.7–100.0)	50.0 (0.0–100.0)	1.3 (0.9–3.0)	0.2 (0.0–1.1)
All^a^	79.9 (73.0–86.0)	83.7 (79.1–87.9)	79.5 (74.0–84.6)	84.2 (78.6–89.3)	4.9 (3.7–6.8)	0.2 (0.2–0.3)

ABPM indicates ambulatory blood pressure monitoring; BP, blood pressure; LR, likelihood ratio; NPV, negative predictive value; PPV, positive predictive value.

a Summary measures are age standardized to the World Health Organization population.

**Table 5:** Interval LRs for Screening Systolic and Diastolic BPs

SBP Interval LRs			DBP Interval LRs		
SBP Interval (mm Hg)	LR Positive	LR Negative	DBP Interval (mm Hg)	LR Positive	LR Negative
<118	0.0 (0.0–0.0)	1.0 (1.0–1.0)	<73	7.3 (2.4–31.4)	0.6 (0.4–0.9)
118–129	7.2 (1.4–39.0)	0.8 (0.6–1.0)	74–80	9.8 (4.5–38.7)	0.3 (0.1–0.6)
130–142	3.1 (1.7–6.1)	0.4 (0.2–0.7)	80–85	4.6 (2.5–10.9)	0.4 (0.2–0.6)
143–159	1.0 (1.0–1.0)	—	86–94	1.6 (1.1–2.3)	0.3 (0.0–0.7)
>160	1.0 (1.0–1.0)	—	>95	1.0 (1.0–1.0)	—

SBP and DBP intervals were determined by dividing the screening blood pressure values into quintiles. Empty cells indicate that there was insufficient data to calculate validity measures. BP indicates blood pressure; DBP, diastolic blood pressure; LR, likelihood ratio; SBP, systolic blood pressure.

## Discussion

This is, to our knowledge, the first population based study in sub‐Saharan Africa that has assessed the validity of screening BP measurements versus ABPM.

In addition to inflating the true prevalence of hypertension in the community by 53% (from 17% to 27%), screening BP measurements failed to identify a significant proportion of the population that was identified as having hypertension using ABPM: Nearly half of the hypertensive participants in this study had masked hypertension. Screening BP measurements would have failed to identify this population. In addition, 4% of the population had white coat hypertension and were at risk of being placed on treatment unnecessarily.^28^, ^29^ Overestimating the total number of patients with hypertension while failing to identify a significant proportion of the population with the condition would lead to inefficient and ineffective use of scarce health resources. Our findings imply that, similar to the situation in developed‐country settings, efforts to control the burden and consequences of hypertension in sSA are likely to be significantly impaired by current screening methods.

The prevailing consensus is that hypertension in developing countries is more prevalent in urban than rural areas^30^; however, data from our study, conducted in rural Kenya, indicate that hypertension is not exclusively an urban disease. This is supported by the previously documented finding of a high burden of stroke and heart failure in the area.^17^ In addition, the recently published 2015 Kenyan Ministry of Health STEPs survey, which used methods similar to the screening strategy employed here, found a 25% prevalence of raised BP in rural areas—remarkably similar to what we found on screening in our study.^31^ The prevalence of raised BP in urban areas in the same (STEPs) survey was 21%. Consequently, lifestyle habits related to urbanization may have a smaller role in elevating BP in African populations than previously thought.

Urinary sodium levels in Kilifi were comparable to those reported by Dahl in Alaskan eskimos in 1958, for which the prevalence of hypertension was zero.^32^ In contrast, results from Nairobi, Kenya, in the Intersalt study found lower median 24‐hour sodium levels of 53 mmol/day, with a 5% prevalence of hypertension,^33^ although this was not age standardized. Mean body mass index levels in our population were well within the normal range. This combination of a high prevalence of hypertension in a rural region with relatively low levels of classic risk factors calls for more detailed study into the pathogenesis of this important condition in tropical Africa.

Previous studies in sSA using ABPM have found high prevalence of the white coat effect among treated hypertensive patients.^34^ Although we found white coat hypertension to be present in 4% of the population, the more significant finding was the high prevalence of masked hypertension in 8% of the population. Given the increased risk of cardiovascular events in this population,^21^, ^35^ strategies aimed at identifying these individuals are urgently needed. The observation that younger age is associated with masked hypertension^36^ may help direct targeted ABPM at this group. It also may be possible to identify individuals with masked hypertension by measuring arterial stiffness indices.^35^

The strengths of this study include its population‐based design, the use of reference standard methods for determining BP, and utilization of a large and representative age‐stratified sample. Limitations include the potential bias introduced by the nested case–control design used, despite demonstration that this approach yields validity results essentially similar to those of full cohort studies.^18^ As expected from the sampling strategy used, participants who underwent ABPM had higher screening systolic BP and were slightly older than the baseline group. Weighting of analyses to correct for the differential probability of undergoing ABPM based on screening results may not have completely eliminated the bias. In addition, a larger proportion of screen‐negative participants had poor‐quality ABPM readings; these limitations mean that although our reported prevalence of masked hypertension was similar to that reported in other studies,^21^, ^37^, ^38^ it may well have been underestimated. The fact that BP measurements were done at home and by nonmedical personnel could also have reduced our ability to detect the white coat effect. Taken together, probable underestimation of masked and white coat hypertension suggest that there could have been more participants who were misclassified using the screening measurement method, possibly strengthening the case for the use of ABPM.

Several issues must be considered before a recommendation to adopt ABPM for diagnosis of hypertension in sSA is made. We observed a modest response rate, with 27% of those referred for ABPM failing to undergo the procedure. This suggests that there may be difficulties regarding acceptability of ABPM in the population. Cost‐effectiveness studies assessing the potential benefit of ABPM in sSA settings are also needed, as are studies to determine whether BP defined by ABPM provides better targeting of BP‐reduction strategies to reduce vascular morbidity.

## Sources of Funding

Etyang, Smeeth and Scott are funded by the Wellcome Trust (Fellowship numbers: 103951/Z/14/Z, 098532 and 098504). The Funders played no role in the preparation of this article.

## Disclosures

None.
